# Immune checkpoint inhibitors as mediators for immunosuppression by cancer-associated fibroblasts: A comprehensive review

**DOI:** 10.3389/fimmu.2022.996145

**Published:** 2022-10-05

**Authors:** Fatemeh Eskandari-Malayeri, Marzieh Rezaei

**Affiliations:** Department of Immunology, School of Medicine, Isfahan University of Medical Sciences, Isfahan, Iran

**Keywords:** cancer-associated fibroblast, inhibitory immune checkpoint, tumor microenvironment, immune suppression, immunotherapy

## Abstract

The tumor microenvironment (TME) is a significant contributor to cancer progression containing complex connections between cellular and chemical components and provides a suitable substrate for tumor growth and development. Growing evidence shows targeting tumor cells while ignoring the surrounding TME is not effective enough to overcome the cancer disease. Fibroblasts are essential sentinels of the stroma that due to certain conditions in TME, such as oxidative stress and local hypoxia, become activated, and play the prominent role in the physical support of tumor cells and the enhancement of tumorigenesis. Activated fibroblasts in TME, defined as cancer-associated fibroblasts (CAFs), play a crucial role in regulating the biological behavior of tumors, such as tumor metastasis and drug resistance. CAFs are highly heterogeneous populations that have different origins and, in addition to their role in supporting stromal cells, have multiple immunosuppressive functions *via* a membrane and secretory patterns. The secretion of different cytokines/chemokines, interactions that mediate the recruitment of regulatory immune cells and the reprogramming of an immunosuppressive function in immature myeloid cells are just a few examples of how CAFs contribute to the immune escape of tumors through various direct and indirect mechanisms on specific immune cell populations. Moreover, CAFs directly abolish the role of cytotoxic lymphocytes. The activation and overexpression of inhibitory immune checkpoints (iICPs) or their ligands in TME compartments are one of the main regulatory mechanisms that inactivate tumor-infiltrating lymphocytes in cancer lesions. CAFs are also essential players in the induction or expression of iICPs and the suppression of immune response in TME. Based on available studies, CAF subsets could modulate immune cell function in TME through iICPs in two ways; direct expression of iICPs by activated CAFs and indirect induction by production soluble and then upregulation of iICPs in TME. With a focus on CAFs’ direct and indirect roles in the induction of iICPs in TME as well as their use in immunotherapy and diagnostics, we present the evolving understanding of the immunosuppressive mechanism of CAFs in TME in this review. Understanding the complete picture of CAFs will help develop new strategies to improve precision cancer therapy.

## Introduction

Cancer is the second leading cause of death worldwide, as one in three people may develop cancer during their lifetime. Intrinsic resistance to classical therapies like chemotherapeutic drugs or radiotherapy remains a key problem in cancer treatments despite efforts and successes in treating cancer over the past few decades. Drug resistance to traditional therapies in solid tumors leads to aggravating problems such as extensive metastasis and neo-angiogenesis, and the spread of cancer throughout the body. Currently, outgrowths of disseminated metastasis remain the primary cause of mortality in cancer patients (1–5).

Tumors can survive and spread because of the interaction between mutant tumor cells and the tumor microenvironment (TME). TME is made up of a complex network that includes an extracellular matrix (ECM), tumor vasculature, adipose cells, fibroblasts and myofibroblasts, neuroendocrine cells, and (about) 50% cancer cells and immune cells (6, 7).

Intracellular communication is controlled by a complicated network of cytokines, chemokines, inflammatory mediators, and matrix remodeling enzymes and uses numerous processes, including direct contact, chemical component production, and signal transduction. The occurrence, growth, metastasis, and drug resistance of cancers are impacted by these mechanisms. Growing evidence shows targeting tumor cells and ignoring the surrounding TME is not effective enough to overcome the cancer disease (5, 8–12).

Cancer-associated fibroblasts (CAFs), which are significant TME elements in the majority of solid tumor types, play a critical role in controlling the biological characteristics of malignancies, including ECM remodeling and tumor spread, treatment resistance, and stimulating angiogenesis. The release of numerous cytokines and chemokines, as well as reciprocal connections that control the recruiting and functional differentiation of innate and adaptive immune cells, are just a few of the ways that CAFs help cancers escape the immune system. As a result, cancer immunotherapy and targeted therapy have focused their attention on CAFs (11, 13–18).

Inhibitory immune checkpoint (iICPs) and their ligands such as T-cell immunoglobulin and mucin-domain containing-3 (TIM-3), programmed cell death-1 (PD-1), cytotoxic T-lymphocyte-associated antigen-4 (CTLA-4), lymphocyte-activation gene- 3 (LAG-3), B and T lymphocyte attenuator (BTLA), and T cell immunoglobulin and ITIM domain (TIGIT) are molecules expressed on the surface of cellular components in the TME, resulting in the suppression of anti-tumor immune responses and eventually leading to tumor survival and progression (18–20). Numerous studies have shown that inhibiting these iICPs and their ligands in various cancers significantly improves and control the disease (21–24).

The role of CAFs in the expression of ligands of various iICPs such as PD-L1 and PD-L2 has been proven in various studies (6, 24). CAFs can also cause the expression of these iICPs at the surface of tumor cells and immune cells in TME and thus result in the suppression of immune cells and, ultimately, tumor growth and progression (18, 25–27).

According to studies, the importance of iICP inhibition as the mainstay of therapy in cancers is very high, and the progression and metastasis of cancer or cancer recovery largely depend on these molecule’s expression patterns (28, 29). Therefore, it can be assumed that at least a part of the positive effect of these targeted therapies is due to their impact on different cells expressing or inducing immunosuppressive inhibitors and their ligands, such as CAFs. In this regard, collecting and categorizing this group of studies is of great importance.

To show new perspectives to offer a new approach to cancer immunotherapy, this review summarizes and categorizes the role of CAFs in iICP expression and/or induction in TME. We will begin the article with an overview of the role of CAFs in immune dysfunction in tumors and then focus on the role of these cells in the expression and/or induction of iICP. Then we will survey therapeutic and diagnostic applications of some potential future treatment modalities based on iICP expression patterns by CAFs.

## Cancer-associated fibroblast as a versatile player in TME

TME is a significant contributor to cancer progression containing complex connections between cellular and chemical components and provides a suitable substrate for tumor growth and development. Among the stromal cells of the TME, CAFs are essential sentinels of the stroma in tumor organs which play a prominent role in the physical support of tumor cells and the enhancement of tumorigenesis (30–32).

Fibroblasts, as non-epithelial and non-immune cells, are the most abundant connective tissue cells almost in all solid organs. These spindle-shaped cells play a role in maintaining tissue integrity under non-cancerous homeostatic conditions. Also, differentiating into myofibroblasts during wound healing lead to tissue repair, synthesis, and regeneration of ECM (33).

Furthermore, fibroblasts can become CAFs due to certain conditions in TME, including tumor-promoting inflammation, oxidative stress, and local hypoxia, and the stimulatory components that adjacent cells release, including transforming growth factor-beta (TGF-β) (34, 35), epidermal growth factor (EGF), platelet-derived growth factor (PDGF) (36), and fibroblast growth factor 2 (FGF2) (33, 36). These essential cells are highly heterogeneous in origin, function, and phenotype and have various endogenous and surface markers, including platelet-derived growth factor receptor (PDGFR)α/β, α-smooth muscle actin (α-SMA), fibroblast activation protein (FAP), podoplanin (PDPN), FSP1 fibroblast specific protein 1 (FSP1). These markers play an essential role in performing their pro-tumorigenic functions by these cells (32, 37–39).

CAFs play a significant role in the solid tumor microenvironment, including carcinomas of the intestine, breast, lung, and pancreas (40–42). The presence of CAFs in TMEs is linked to a poor prognosis and treatment resistance, angiogenesis and metastasis, disease recurrence, immunosuppression, ECM remodeling, and metabolic reprogramming (43, 44). For example, a study conducted in 2021 on CAFs in four histological types of lung adenocarcinoma, it was stated that the presence of CAF cells is associated with a poor prognosis of patients (45). Also during another study on esophageal squamous cell carcinoma (ESCC) it was found that the density of CAFs can be a marker to predict the prognosis and guide the treatment process in ESCC (46). Moreovere, higher CAF density within the head and neck squamous cell carcinoma (HNSCC) microenvironment is associated with advanced T stage, nodal infiltration, clinical stage, vascular invasion, perineural invasion, differentiation, increased rates of local recurrence and ultimately poor prognosis in HNSCC (47). Studies have shown that CAFs perform these roles through the secretion of growth factors, production of matrix metalloproteinases (MMP), cytokines, and chemokines which cause the induction of angiogenesis, cancer cell proliferation, and tumor-enhancing inflammation and finally lead to tumor progression and invasion (15, 48, 49).

Through a variety of direct and indirect processes on specific immune cell populations, such as the secretion of numerous cytokines and chemokines, interrelations that facilitate the recruitment of regulatory immune cells, and the reprogramming of an immunosuppressive function in immature myeloid cells, CAFs are also emerging as a key component in immune regulation that forms and modulate the immune response in TME. This contributes to the immune escape of tumors. Additionally, CAFs directly inhibits the activity of cytotoxic lymphocytes by killing CD8+T cells in an antigen-dependent manner *via* PD-L2 and FASL (50) and increasing the synthesis of matrix components such as hyaluronan Q16 (14, 15) and change the T cell role in the TME to one that promotes tumor growth (15). In terms of their inhibitory effects, various studies have shown that CAFs could be an origin of iICPs and their ligands and also can induce their expression of them at the surface of tumor cells and immune cells (27).

## Inhibitory immune checkpoints (iICP) in TME

To persist and develop cancer, cancer cells must avoid immune detection and death *via* cytotoxic lymphocytes. This immunosuppressive milieu in TME is shaped by mechanisms such as decreased antigen presentation, increased surface inhibitory molecules expression, and release of immunosuppressive substances (15).

The presence of active and functional immune cells in TME, especially T lymphocytes, is associated with good prognosis, disease control, and even tumor recovery. T cell activation is regulated by co-stimulatory and co-inhibitory receptors, known as immune checkpoints (ICPs). Mostly, T cells in the TME have the exhausted and dysfunction phenotype. These conditions occur following a progressive loss of effector function, expression of multiple co inhibitory receptor, downregulation activity of stimulatory immunoreceptors and a common transcriptional and epigenetic program (51–53). One of the most important cells that can create the mentioned conditions and cause exhausted T cells are CAF cells. In addition to creating exhausted T cells, in a study conducted on patients with CRC, it was shown that the presence of CAF cells leads to the recruitment and migration of Treg lymphocytes and significantly increases the frequency of these inhibitory cells in the tumor environment (54). On the other hand, during another study on pancreatic ductal adenocarcinoma (PDAC), it was stated that exhaustion of myofibroblasts in this cancer leads to increased proliferation of CD4+ Foxp3+ Tregs in the tumor environment and inhibits the immune system in the tumor environment (50).

The most well-known iICPs are classified into three prominent families: B7-CD28 superfamily like PD-1, PDL-1, PDL-2, CTLA-4, BTLA, B7H3 (55), TNF superfamily likes HVEM (52, 56), and immunoglobulin superfamily likes PVR, LAG-3, TIM-3, TIGIT, VISTA, HLA-E (52).

Considering the inhibitory effects of iICPs on immune responses, they are essential for preserving self-tolerance as well as regulating the intensity and duration of immune response effectors in various tissues to minimize tissue injury. Most of the iICPs are expressed on cells of the innate immune system like antigen-presenting cells (APCs), as well as the adaptive immune system cells, specifically T cells. Evidence has shown that iICP molecules and their ligands are over-expressed on tumor cells and stromal cells (CAFs) in TME. In addition to immunosuppression as the primary function of iICPs, it has been reported that these molecules are crucial for maintaining a variety of malignant characteristics, such as self-renewal, epithelial-to-mesenchymal transition, metastasis, drug resistance, anti-apoptotic activity, angiogenesis, and improved energy metabolisms (57, 58).

iICPs and their ligands as immunosupressive agents have improved cancer therapy and increased our understanding of anti-cancer immunity. Current clinical trials have introduced cancer immunotherapy using different iICPs and their ligands, such as monoclonal antibodies (mAbs) against CTLA-4, PD-1 or PD-L and, Tim-3 (29, 59–69). For example, ipilimumab, a mAb that targets CTLA-4, was certified as a iICP inhibitor in order to treat metastatic melanoma, MSI-H/dMMR CRC, and renal cell carcinoma (RCC). Also, Nivolumab, Pembrolizumab, and Cemiplimab, are the mAbs that targets PD-1 and approved in order to treat MSI-H or dMMR CRC, HNSCC, hepatocellular carcinoma (HCC). Moreover, NCT00349934 and NCT02614833, are mAbs that target Tim-3 in order to treat breast cancer (BC) was certified as a iICP inhibitor (29, 53, 60, 64, 67, 68, 70). The restoration of tumor-specific immunity due to the inhibition of iICPs revitalizes malfunctioning T cells and helps tumor cells be eradicated (29, 67, 68, 71).

## Inhibitory immune checkpoints and cancer-associated fibroblast

Given that the tumor stroma in TME can inhibit T-cells, it is possible that CAF-derived mediators can alter the phenotype and activities of T-cells (72). Alternatively, activated CAFs can secrete factors that serve or induce the expression of iICP and their ligands on the different cells in TME. This note is essential about the tumors that their stroma is dense and dominant with activated CAFs. The exhausted T-cells that express iICP molecules lose proliferative potential and cytotoxicity ability.

**Figure 1:**
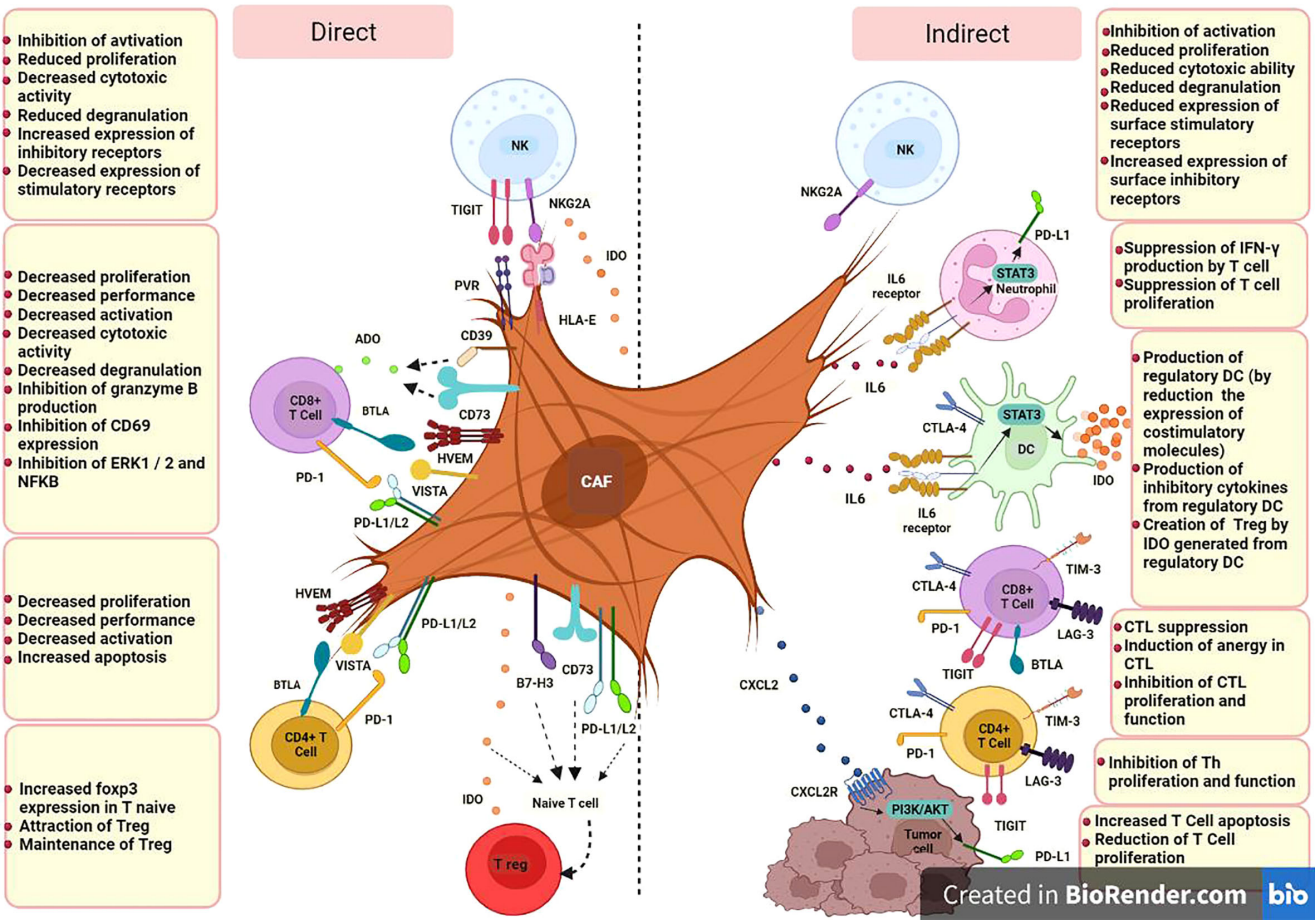
CAF cells could modulate immune cell function in TME through iICPs in two ways; direct expression of inhibitory membrane-associated molecules and indirect induction of them by producing soluble factors. CD8+ T cell, Cytotoxic T-cell (CTL);CD4+ T cell, Helper T-cell (Th); DC, Dendritic Cell; NK, Natural killer cell; T reg, Regulatory T cell; PD-L1, Programmed Cell Death Ligand 1; PD-L2, Programmed cell death-ligand 2; IDO, Indoleamine 2;3 dioxygenase; ADO, Adenosine; HLA-E, human leukocyte antigen E; PVR, poliovirus receptor; A2B, adenosine A2B receptor; NKG2A, CD159a; TIM-3, T-cell immunoglobulin and mucin-domain containing-3; PD-1, programmed cell death; CTLA-4, cytotoxic T-lymphocyte-associated antigen-4; LAG-3, lymphocyte-activation gene- 3; BTLA, B and T lymphocyte attenuator; TIGIT, T cell immunoglobulin and ITIM domain; HVEM, herpes virus entry mediator; VISTA, V-domain immunoglobulin suppressor of T cell activation; B7-H3, CD276; CD, Cluster of Differentiation; IL, Interleukin; CXCL, C-X-C Motif Chemokine Ligand; CXCR, C-X-C chemokine receptor; STAT3, Signal transducer and activator of transcription 3; IFN-γ, Interferon-gamma.

Based on available studies, specific CAF subsets which can be identified in terms of markers panel, origin and function, could modulate immune cell function in TME through iICPs in two ways; direct expression of inhibitory membrane-associated molecules, e.g., PD-1 or LAG3, by activated CAFs and indirect induction of them by producing soluble factors, e.g., transforming growth factor β (TGF-β) or IFN-γ inducible protein-10 (IP-10, also known as CXCL10) (**Figure 1**).

## Direct expression of iICPs by cafs

Normal fibroblasts (NFs) in diverse tissue have a primary expression of iICPs. Sometimes this primary expression is proportional to the function of these cells. For example, CD90+ myofibroblasts, as the significant cell phenotype in the normal human colonic lamina propria, function as the nonprofessional MHC class II+ antigen-presenting cells adjusted to epithelial cells. These stromal cells constitutively express PD-L1 and PD-L2 molecules and, through regulation of the acute pro-inflammatory responses of activated T helper cells, may be involved in mucosal tolerance. After chronic stimulation in TME, the expression of iICPs is increased by CAFs and prevents immune responses against the tumor and leads to growth and progression and tumor metastasis (73).

(**Table 1**) comprehensively listed the direct role of CAFs on the modulation of the immune cells in TME by the expression of iICPs. CAFs can impact the innate and adaptive immune cells in TME, but since T-cells are the predominant immune cell subtypes with a high potential to eliminate cancer cells., we first focused on the CAF-T cell interactions through iICP signaling.

**Table 1:** List of the induced or over-expressed iICP and their ligands on CAFs in different tumors.

ICP Superfamily	Tumor Type	Type of Analysis	Type of iICP	Origin of samples	CAF subtypes	Result of Study	Ref
**B7-CD28 superfamily**	NSCLC	FC	PD-L1 PD-L2	NSCLC tumor tissues	SM1214P^+^ (HLA-A, -B, -C) ^+^ Thy1^+^ FAP^+^ α-SMA^+^	CAFs in human NSCLC are functionally and phenotypically heterogeneous and provide multiple complex regulatory signals that have the potential to enhance or suppress CAF function in the TME.	(72)
	Melanoma	FC	PD-L1 PD-L2	lymph nodes, soft tissue, lung, brain, and chest wall	α-SMA^+^	IL-1 inhibition by vemurafenib leads to PDL 1 and PDL 2 downregulation at the CAF level.	(18)
	HNSCC	FC	PD-L1 PD-L2	HNSCC tumor tissues	CD90^+^ FAP^+^ α-SMA^+^	CAFs, unlike NFs, expressed PD-L1 and PD-L2, and the expression levels of cytokine genes, including IL6, CXCL8, TNF, TGFβ1, and VEGFA, were higher in CAFs. CAFs and their supernatant are stronger than NFs in suppressing T cell proliferation, T cell apoptosis, and inducing regulatory T cells.	(74)
	Melanoma and lung cancer	FC	PD-L2	Tumor-bearing mice	PDPN^+^ PDGFRα^+^ PDGFRβ^+^ FAP-α^+^	CAFs, kill CD8+T cells in an antigen-dependent manner *via* PD-L2 and FASL	(75)
	OvC	FC	B7-H3	OvC tumor tissue	PDGFRβ^+^FAP^+^ high	On non-immune cells, B7-H4 expression was restricted to tumor cells, whereas B7-H3 was expressed by both tumor and stromal cells. Stromal cells of the ovarian TME expressed higher levels of B7-H3 compared to tumor cells.	(76)
	PC	FC	PD-L1 PD-L2	PC tumor tissues	CD29^+^, CD44^+^, CD73^+^, CD90^+^, CD105^+^ ICAM-1^+^, HLA class I^+^ α-SMA^+^, FAP^+^ PDPN^+^	CAFs expressed higher levels of the PD-L1 and PD-L2 compared to primary skin fibroblasts from healthy donors.	(27)
	NSCLC	qRT-PCR FC IHC	PD-L1	NSCLC tumor tissues	α-SMA^+^	In the non-metastatic NSCLC, PD-L1 expression on CAFs is reversibly regulated by IFN-γ from activated lymphocytes and suggests the induction of anti-tumor immune responses, contributing to a better prognosis after surgery.	(77)
	TNBC	IHC TMA Double-IF	PD-L1	TNBC tumor tissues	α-SMA^+^	PD-L1 expression by CAF is a novel marker for a better prognosis for patients with TNBC, IHC may be suitable for immunostaining of PD-L1^+^ CAF.	(78)
**TNF superfamily**	Melanoma	FC	HVEM	Melanoma tissues	FAP^+^ Melan-A^-^ gp100^-^	Compared to Dermal fibroblasts, CAFs displayed increased amounts of HVEM, a known ligand of BTLA on T cells, increased l-arginase activity, and CXCL12 release. There is also an increase in the expression of TIGIT and BTLA in CD45RO+ non-naïve/memory cytotoxic T cells following exposition to CAF in comparison to Dermal fibroblast.	(20)
**Ig superfamily**	Melanoma	FC	VISTA	Human Melanoma tissues	FAP^+^ Melan-A^-^ gp100^-^	Compared to Dermal fibroblasts, CAFs displayed increased amounts of VISTA, a known ligand of BTLA on T cells, increased l-arginase activity, and CXCL12 release. There is also an increase in the expression of TIGIT and BTLA in CD45RO+ non-naïve/memory cytotoxic T cells following exposition to CAF in comparison to Dermal fibroblast.	(20)
	NSCLC	FC	PVR HLA-E	NSCLC tumor tissues	α-SMA^+^ FAP^+^	CAFs inhibit NK cell activation by reducing their proliferation rates, the cytotoxic capacity, the extent of degranulation, and the surface expression of stimulatory receptors, while concomitantly enhancing the surface expression of inhibitory receptors in NK. There was a significant increase in NKG2A expression level in the irradiated CAF group compared to the NF group. Irradiated CAFs, compared to radiation-free CAFs, have enhanced expression of iICP ligands such as PVR and HLA-E	(19)
**Others**	HCC	WB	IDO	HCC tumor tissues	a-SMA^+^ FAP^+^ Fn^+^ FSP^+^ VIM^+^ DES^+^	PGE2 and IDO, derived from CAFs, suppress the activation of NK cells and thereby create favorable conditions for tumor progression.	(79)
	CeCa	FC IHC UPLC	CD39/CD73 ADO	CAFs from MSCs of normal CeCa tissues	CD105^+^ CD90^+^ CD73^+^ (HLA-ABC)^+^ low	Higher expression of levels of CD39 and CD73 by CeCa−MSCs compared to NCx−MSCs was associated with the ability to suppress the proliferation, activation, and functions of cytotoxic T−cells through the generation of large amounts of Ado by CeCa−MSCs.	(80)
	CRC	IF qRT-PCR FACS IHC	CD73 A2B	Tumor-bearing mice	PDGFRα^+^ PDGFRβ^+^ FAP^+^ Acta2^+^ DES^+^ Postn^+^ Thy1^+^	CAFs constitute the prominent CD73hi population in human CRCs and two CD73−murine tumor models, including a modified CRC. High CAF abundancy in CRC tissues correlates strongly with elevated CD73 activity and poor prognosis. CAF-CD73 expression is enhanced *via* an ADO-A2B receptor-mediated feed-forward circuit triggered by tumor cell death, which enforces the CD73 checkpoint.	(81)

NSCLC, Non-small cell lung cancer; HNSCC, Head and neck squamous cell carcinoma; PC, pancreatic cancer; OvC, Ovarian cancer; TNBC, Triple-negative breast cancer; HCC, Hepatocellular carcinoma; CRC, Colorectal cancer; CeCa, Cervical cancer; IHC, Immunohistochemistry; IF, Immunofluorescence; FC, Flow cytometry; qRT-PCR, Quantitative real-time reverse-transcription PCR; WB, Western blot; UPLC, Ultra-performance liquid chromatography; FACS, Fluorescence-activated cell sorting; TMA, tissue microarray; PD-L1, Programmed Cell Death Ligand 1; PD-L2, Programmed cell death-ligand 2; IDO, Indoleamine 2,3 dioxygenase; HLA-E, human leukocyte antigen E; PVR, poliovirus receptor; A2B, adenosine A2B receptor; B7-H3, CD276; CD, Cluster of Differentiation; α-SMA, Smooth muscle alpha-actin; ICAM-1, Intercellular adhesion molecule-1; FAP, Fibroblast-activation protein; PDPN, Podoplanin; PDGFR, platelet-derived growth factor receptor; HLA, Human leukocyte antigen; Thy1, CD90; Fn, Fibronectin; FSP1, Fibroblast-specific protein 1 (also called S100A4); VIM, Vimentin; Acta2, αSMA; DES, Desmin; Postn, Periostin; Melan-A, melanoma antigen recognized by T cells 1 or MART-1; Gp100, Glycoprotein 100; IL, Interleukin; TGFβ, Transforming growth factor beta; IFN-γ, Interferon-gamma; CXCL, C-X-C Motif Chemokine Ligand; VEGFA, Vascular endothelial growth factor A; B7-H4, coinhibitory molecule belongs to B7 family; FASL, Fas ligand/CD95L; TNF, Tumor necrosis factor; PGE2, Prostaglandin E2; CeCa−MSCs, Cervical cancer-derived mesenchymal stromal cells; NCx−MSCs, Normal cervix mesenchymal stromal cells; NKG2A, CD159a.

Different studies showed that CAFs in tumors such as pancreatic ductal adenocarcinoma (PDAC) or non-small cell lung cancer (NSCLC) have high expression levels of PD-1 ligands, so it could be conceivable that CAFs affect T cell functionality following PD-L1/L2 binding (24, 82). In some cases, it is even called the primary PD-L1/PD-L2+ cells are CD90+ CAFs (73). In the survey of the suppressive effect of CAFs on the T cell function in TME, treatment of CAFs separated from head and neck squamous cell carcinoma tumors with anti-PD-L1/L2 mAbs and co-culture with T cells significantly restored T cell proliferation. These findings confirmed that co-regulatory molecules play a role in the inhibitory effects of CAFs (74).

Immunohisto-staining of the different tumor tissues steadily reveals that more than 90% of all T cells in the TME are located in the stroma, where they are close enough to potentially interact with cancer cells and as well as CAFs. In this regard, Laia Gorchs and colleagues in a study on human PDAC tissues showed T cells in TME were near α-SMA+ CAFs in PDAC tissue. Their results indicated that PD-1+ Tim-3+ T cells separated from PDAC tumors had reduced functionality in the presence of CAFs, including lower level expression of CD107a and inflammatory cytokines, e.g., IFN-γ and TNF-α. It was also shown that the expression of α-SMA, podoplanin, PD-L1, and PD-L2 on CAFs is not due to changes in different passages but to the conversion of these cells from normal to the active state (27).

Elevated levels of PD-L2 gene expression were validated in pancreatic, colon, and breast malignancies using microarray data sets (RNA expression) of CAFs from human tumor tissues and normal counterparts. In accordance with data from *in vivo* and *in vitro* experiments, PD-L2 is a prevalent iICP expressed by CAFs and thorough direct interactions with T cells, in line with other inhibitory molecules such as FASL, derived specific deletion of CD8+ T cells in an antigen-specific manner (75).

Despite the constant and high expression of these iICPs by activated CAFs, these expressions are significantly different depending on the tumor type or even between patients. For example, the study on the CAFs isolated from NSCLC human samples revealed PD-L1 expression ranged from 63-96%, and for PD-L2, the range was 13–82%. The data of this experiment also showed the expression of these two inhibitory ligands is affected by some stimuli such as environmental cytokines because it was confirmed that the addition of IFN-γ to the culture medium increased the expression of PD-L1/L2 in CAFs (72). Given that activated lymphocytes are the main source of IFN-γ in tumor tissues, in another study, it was assessed the effect of lymphokine-activated killer cells secreting IFN-γ on the PD-L1 expression in co-culture with CAF cells.The findings demonstrated that PD-L1 expression on CAFs was up-regulated in this interaction. According to these data can be inferred that the presence of PD-L1+ CAFs in tumor tissues may signify that T cells that release IFN have been activated and have infiltrated the TME (77).

In addition to the inhibitory effects of CAFs on T cells by iICPs signaling, CAFs interacted with other cells, such as the natural killer cell (NK) in TME, too. By lowering NK cell proliferation and cytotoxic potential, CAFs prevent NK cell activation while at the same time enhancing overexpression of inhibitory receptors on their surface, as an essential inhibitory mechanism that prevents NK cell activation (19).

As well as in connection with the direct inhibitory function of PD-1 ligands in the TME, it is approved that the expression of these inhibitory ligands by CAFs is associated with tumor prognosis (78). Koji Teramoto et al. using tumor samples obtained from specimens of patients with NSCLC showed the importance of the PD-L1+ CAF population in the prognosis of this tumor. Their IHC results on tumor tissue samples showed relapse-free survival after surgery in NSCLC patients with PD-L1+ CAF was higher than in others (77). Furthermore, it has been specified that the presence of PD-L1+ CAF in tissues obtained from patients with triple-negative breast cancer (TNBC) was associated with a better prognosis, too (78).

CAF subsets in TME characterize by distinct properties and levels of activation and already different CAF populations with heterogenic functions recognized in various tumors such as breast or pancreas. Based on six approved markers, it was highlighted the existence of at least four CAFs subtypes in breast cancer. Between maintained cells, CAF-S1 with CD29Med, FAPHi, FSP1Med, α-SMAHi, PDGFRβMed-Hi, CAV1Low phenotype is the prominent subset in aggressive (HER2 and TN) BC. CAF-S1 attracts T lymphocytes, increases the survival of CD4+CD25+ T lymphocytes, and promotes their differentiation into CD25+FOXP3+ cells. CAF-S1 also improves the ability of Treg cells to suppress effector T cell growth and proliferation. Moreover, to OX40L, PD-L2 has a role in the retention of Treg cells *via* CAF-S1 cells. So CAF-S1 by expression of PD-L2 could be a source of resistance to immunotherapy strategies that target PD-L1 (83).

The study on human tissue samples of ovarian cancer demonstrated that B7 family proteins had different expression patterns in TME. In the ovarian TME, for example, both tumor and stromal cells expressed B7-H3, although stromal cells have higher surface levels of B7-H3 than do tumor cells. On the other hand, they discovered that the expression of the B7-H3 protein was closely related to the expression of wild-spread biomarkers of CAF; FAP, and PDGFRβ. This association indicates that B7-H3 expression is mainly related to CAFs than other cancer-associated stromal cells. Consistent with this, a restricted expression pattern was seen for PD-L1. It did not co-express, for example, with B7-H4 and was primarily expressed on stromal cells in the ovarian TME. Due to the varied expression pattern, it is possible that combination therapy against both molecules may affect distinct cell populations in the ovarian TME that have the potential to have additive or synergistic effects. Furthermore, the data of this experiment suggested that stromal content (tumor to stroma ratio) may impact disease outcome, as patients’ poor overall and disease-free survival has been linked to increased stromal tumor content. This may be due to where high stromal content was present, immune cell activation restricted by them. As a result of the differential expression pattern of these iICPs in the stroma and tumor cells and the importance of tumor to stroma ratio as a prognostic factor, designing a model based on these markers’ expression patterns could be helpful as a diagnostic test for defining the phenotype of infiltrating immune cells and managing the ovarian cancer immunotherapy (76).

Adenosine (Ado) is a potent extracellular, most important immunosuppressive messenger in the TME (84). Dramatically, in stressful conditions like hypoxia, in a multistep coordinated cascade, produced high concentrations of Ado and, by binding to its activated receptors, influence biological pathways in tumor cells and immune cells, and thereby affect tumor progression and immunosuppression, respectively (85). CD73, as a crucial ICP is a glycosylphosphatidylinositol (GPI) anchored cell surface protein that, along with other ectonucleotidases, the most important of them is CD39, is involved in the production of large amounts of Ado and cell interaction with ECM components and as a result, mediate cancer invasive and metastatic (86).

One of the essential sources of CD73 in TME is CAF. Comparative investigations using transcriptome datasets from human CRC tissues, resected CRC specimens and CAFs isolated from human tissue and mouse tumor models revealed that CAFs are the outstanding CD73^hi^ cells in the TME and have higher bioactivity in comparison with other cellular residents within the TME. On the other hand, elevated CAF abundance and immunosuppression are also linked to high CD73 levels in the TME of human CRCs (81).

The overexpression of CD73 in solid tumors has been found to promote tumor progression by enhancing tumor cells’ invasiveness and suppressing anti-tumor immunity (87). This is partly due to the non-enzymatic features of CD73. *In vitro* experiments on the epithelioid sarcoma cell line, FU-EPS-1, and ST353i as human dermal fibroblast demonstrated an interaction between CD73 on fibroblast and CD147 (extracellular matrix metalloproteinase inducer, an imprint), performs a crucial part in controlling how much MMP2 is produced by activated fibroblasts. MMP-2 (Gelatinase A), the TME’s most prevalent MMP promotes tumor invasion. Anti-CD73 neutralizing antibodies were added to co-cultured cells, and this decreased MMP-2 production in the supernatant, indicating the importance of the non-enzymatic activity of CD73 in regulating MMP production and ECM remodeling (88).

Indeed, high levels of CD73 expression by CAF promote cancer cells proliferation (86) and are associated with poor prognosis in different cancers like CRC (81), prostate cancer (89), Squamous cell carcinoma of the external auditory canal (SCC-EAC) or nonmuscle invasive bladder cancer (89, 90).

In muscle-invasive bladder cancer (MIBC) patients, CD73+ stromal fibroblasts in their tumors are an approximately twofold non-invasive group. The critical point is that the high presence of CD73+ CAFs in the MIBC cohort correlated with tumor grade and poor progression-free survival (87). A similar finding was demonstrated compared to normal cervical tissue-derived MSCs, and cervical cancer-derived MSCs have greater amounts of CD39 and CD73 ectonucleotidases in cell membranes. This characteristic was linked to Ado’s potent capability to inhibit the proliferation and effector functions of cytotoxic T-cells *via* the high-affinity Ado receptor A2A. Ado produced by MSCs decreased CD8+ T-cell proliferation, IFN- production, and the CTL antigen-specific effector capacity in a dose-dependent manner (87).

To other iICP expressions by CAFs and their possible role in CAF-mediated immunosuppression, Barbara Érsek and colleagues 2020 compared the expression of CAF-iICPs on melanoma-associated fibroblasts (MAFs) and dermal fibroblasts (DFs) isolated from melanoma patients and skin biopsies, respectively. They evaluated the expression and presence of different iICP ligands, including PD-L1, Galectin-3, Galectin-9, CD155, Herpesvirus entry mediator (HVEM), and V-domain Ig suppressor of T cell activation (VISTA). Compared to DFs, MAFs showed elevated levels of the negative CTL regulators VISTA and HVEM. These ligands at the surface of MAFs bind to their receptors at the surface of CTL, leading to CTL anergy (20).

## Indirect upregulation of iicp by cafs

Following activation of CAFs in the TME, it can orchestrate the recruitment of peripheral immune cells towards the tumor and affect the differentiation and activation of infiltrated cells through the secretion of a wide variety of cytokines, chemokines, and growth factors (24).

Although the CAFs secretome is still not thoroughly characterized as a significant immunomodulator in the tumor stroma, CAFs can trigger specific signaling pathways in tumor and immune cells that lead to immune evasion and tumor progression. Accumulating evidence suggests that one of the essential inhibitory mechanisms driven by CAFs subtypes is the induction of inhibitory ICPs and their ligands on tumor and immune cells (27) (**Table 2**).

**Table 2:** Induction of iICPs and their ligands on the cell surface in TME by CAFs.

ICP Superfamily	Tumor Type	Type of Analysis	Type of iICPs	Origin of samples	CAF subtypes	Target cell	Immune modulators secreted by CAFs	Result of Study	Ref
B7-CD28 superfamily	HCC	FC WB	CTLA4	The foreskin of patients	———	DC	IL-6		(91)
	Melanoma and lung cancer	FC	PD-1	Tumor-bearing mice	PDPN^+^ PDGFRα^+^ PDGFRβ^+^ FAP-α^+^	CD8+T cells	———–	CAFs support T cell suppression within the TME by a mechanism dependent on ICP activation.	(75)
	HCC	FC	PD-L1	Human hepatitis B-related HCC tissues	α-SMA^+^	Neutrophils	SDF1a IL-6	HCC-CAFs attract peripheral blood neutrophils through the SDF1a/CXCR4 pathway. HCC-CAF-derived IL-6 was responsible for the STAT3 activation of neutrophils. Following STAT3 activation, PDL1 is expressed at the surface of the neutrophil. Then neutrophils impaired T-cell function through the PD1/PDL1 signaling pathway.	(25)
	LUAD	qRT-PCR IHC	PD-L1	Human LUAD tumor tissues	α-SMA^+^	Tumor cells	CXCL2	CXCL2 produced by CAFs increases the potential to induce PD-L1 expression in lung adenocarcinoma cells.	(92)
	PC	FC	PD-1 CTLA-4	PC tumor tissues	CD29^+^, CD44^+^, CD73^+^, CD90^+^, CD105^+^ ICAM-1^+^, HLA class I^+^ α-SMA^+^, FAP^+^ PDPN^+^	T cell	COX-2 PGE2	CAFs promoted the expression of TIM-3, PD-1, CTLA-4, and LAG-3 in proliferating T-cells and contributed to a diminished immune function. CAFs strongly inhibited T-cell proliferation in a contact-independent fashion.	(27)
	Melanoma and CRC	IHC FC qRT-PCR WB	PD-L1	Primary murine CAFs isolated from subcutaneous tissues	α-SMA^+^	Tumor cells	CXCL5	LY294002, the inhibitor of PI3K, confirmed that CXCL5 derived by CAFs created an immunosuppression microenvironment by promoting PD-L1expression in tumor cells *via* PI3K/AKT signaling.	(93)
	BC	FC WB	PD-L1	Human BC tumor tissues	FSP1^+^ VIM^+^ α-SMA^+^	Tumor cells	CAF-derived exosomes	CAF-derived exosomes promote miR-92 expression in BC cells. miR-92 targets LATS2 and enhance the nuclear translocation of YAP1. The nuclear translocation of YAP1 leads to increased transcription and expression of PD-L1 in breast cancer cells. After treatment of BC cells by CAF-derived exosomes, cancer cells express higher PD-L1.	(94)
	Melanoma	FC	BTLA	Human Melanoma tumor tissues	FAP^+^ Melan-A^-^ gp100^-^	T cell	Arginase	The expression of arginase in CAFs increased BTLA and TIGIT on CD8+ T cells. CAF interferes with intracellular CTL signaling *via* soluble mediators leading to CTL anergy. Increased expression of TIGIT and BTLA in CD45RO+ non-naïve/memory cytotoxic T cells following exposition to CAF as compared to Dermal fibroblast.	(20)
	BC NSCLC	FC	PD-1 CTLA4	Human BC tumor tissues	CAF-S1 (ecm-myCAF): FAP-hi α-SMA hi CD29 med-hi MCAM low ANTXR1^+^ SDC1^+^ LAMP5^−^ CD45^−^ EPCAM^−^ CD31^−^ CD235a^−^	CD4+ T cells	————	Cluster 0/ecm-myCAF upregulates PD-1 and CTLA4 protein levels in Tregs, which, in turn, increases CAF-S1 cluster 3/TGFβ-myCAF cellular content.	(95)
	Lung cancer CRC BC	RNA-Seq FC IHC	CTLA-4	Tumor-bearing mice	α-SMA^+^	CD8+T cells	NOX4 enzyme	RNA sequencing of CD8+T cells from CAF-rich murine tumors and immuno-chemistry analysis of human tumors identified significant up-regulation of CTLA-4 in the absence of other exhaustion markers; NOX4 inhibition restored immunotherapy response in CAF-rich tumors.	(96)
Ig superfamily	PC	FC	TIM-3 LAG-3	Human PC tumor tissues	CD29^+^ CD44^+^ CD73^+^ CD90^+^ CD105^+^ ICAM-1^+^ HLA class I α-SMA^+^ FAP^+^ PDPN^+^	T cell	COX-2 PGE2	CAFs strongly inhibited T-cell proliferation in a contact-independent fashion. CAFs promoted the expression of TIM-3, PD-1, CTLA-4, and LAG-3 in proliferating T-cells	(27)
	Melanoma	FC	TIGIT	Human Melanoma tumor tissues	FAP^+^ Melan-A^-^ gp100^-^	T cell	Arginase	The expression of arginase in CAFs increased BTLA and TIGIT on CD8+ T cells. CAF interferes with intracellular CTL signaling *via* soluble mediators leading to CTL anergy. Increased expression of TIGIT and BTLA in CD45RO+ non-naïve/memory cytotoxic T cells following exposition to CAF as compared to Dermal fibroblast.	(20)
	BC NSCLC	?????	TIGIT	Human BC tumor tissues	CAF-S1 (ecm-myCAF): FAP-hi α-SMA hi CD29 med-hi MCAM low ANTXR1^+^ SDC1^+^ LAMP5^−^ CD45^−^ EPCAM^−^ CD31^−^ CD235a^−^	CD4+ T cells	————	Analysis of more than 19,000 single CAF-S1 fibroblasts from breast cancer identified 8 CAF-S1 clusters. Myofibroblasts from clusters 0 and 3, characterized by extracellular matrix proteins and TGFβ signaling, respectively, are indicative of primary resistance to immunotherapies. Cluster 0/ecm-myCAF upregulates PD-1 and CTLA4 protein levels in Tregs, which, in turn, increases CAF-S1 cluster 3/TGFβ-myCAF cellular content.	(95)
Others	HCC	FC WB	IDO	The foreskin of patients	—————	DC	IL-6	CAFs derived from HCC tumors facilitate the generation of regulatory DCs, which are characterized by low expression of costimulatory molecules, high suppressive cytokine production, and promotion of Treg expansion *via* IDO upregulation. STAT3 activation in DCs, as mediated by CAF-derived interleukin IL-6, is essential to IDO production from DCs	(91)
	NSCLC M26	FC	NKG2A	Human NSCLC tumor tissues	FAP-1^+^ a-SMA^+^	NK cells	———	There was a significant increase in NKG2A expression levels in NK cells exposed to the CAF and iCAF group compared to the Normal fibroblast group.	(19)

NSCLC, Non-small cell lung cancer; PC, pancreatic cancer; HCC, Hepatocellular carcinoma; CRC, Colorectal cancer; LUAD, Lung adenocarcinoma; BC, Breast cancer; IHC, Immunohistochemistry; FC, Flow cytometry; qRT-PCR, Quantitative real-time reverse-transcription PCR; WB, Western blot; RNA-seq, RNA-sequencing; PD-L1, Programmed Cell Death Ligand 1; IDO, Indoleamine 2;3 dioxygenase; NKG2A, CD159a; CD, Cluster of Differentiation; α-SMA, Smooth muscle alpha-actin; ICAM-1, Intercellular adhesion molecule-1; FAP, Fibroblast-activation protein; PDPN, Podoplanin; PDGFR, platelet-derived growth factor receptor; HLA, Human leukocyte antigen; FSP1, Fibroblast-specific protein 1 (also called S100A4); VIM, Vimentin; Melan-A, melanoma antigen recognized by T cells 1 or MART-1; Gp100, Glycoprotein 100; IL-6, Interleukin 6; TGFβ, Transforming growth factor beta; SDF1, Stromal cell-derived factor 1; CXCL, C-X-C Motif Chemokine Ligand; COX-2, Cyclooxygenase-2; PGE2, Prostaglandin E2; NOX4, NADPH oxidase 4; CXCR, C-X-C chemokine receptor; miR-92, MicroRNA 92; LATS2, Large tumor suppressor kinase 2; YAP1, yes-associated protein 1; STAT3, Signal transducer and activator of transcription 3.

In confirmation of this, Laia Gorchs et al. in-depth examination using CAFs isolated from desmoplastic pancreatic tumor tissues collected from PDAC patients demonstrated that CAF-derived factors induce expression of iICPs on CD4+ and CD8+ T-cells and may be able to hold back T-cell functions in TME. This is in line with other observations in different tumor types such as CRC, breast cancer, lung cancer (39), or melanoma (75). In this regard Gorchs L in 2019 showed that T-cells residing within the desmoplastic stromal compartment of PDAC tissue express PD-1 and TIM-3, indicating a role for CAFs on co-inhibitory marker expression *in vivo*. It was also revealed that CAFs strongly inhibited T-cell proliferation in a contact-independent fashion. By transwell assay compared to direct co-cultures of PBMC and pancreatic CAFs, they showed the up-regulation of co-inhibitory markers such as TIM-3, LAG-3, and CTLA-4 on T-cells was at least partially mediated by CAFs-derived soluble factors in a dose-dependent manner. According to their data, prostaglandin E2 (PGE2) is the essential factor involved in the increased co-expression of TIM-3 and PD-1, as that blocking of the activity of PGE2 produced by CAF with indomethacin partially restored the proliferative capacity of both CD4+ and CD8+ T-cells. PGE2 is the major metabolite of the COX-2 pathway that, as previously approved, expressed by CAFs in different tumor types (27, 97, 98). Also, in the other study showed a high amount of PGE2 produced by CAFs leads to anti-tumor immunity suppression such as NK dysfunction and promotes tumor growth and survival (79).

Further, Ziqian Li using primary murine CAFs isolated from the subcutaneous tissues and different mouse and human cell lines, stated that CXCL5 produced by CAFs leads to the expression of PD-L1 on the surface of melanoma and CRC tumor cells. A series of experiments and treatment of cancer cells with the small molecular inhibitor LY294002 as an inhibitor of PI3K/AKT signaling indicated that CXCL5 secreted by CAFs binds to the CXCR2 receptor at the surface of tumor cells and induces PD-L1 expression at these cells *via* this pathway (93).

Mediators secreted by CAFs can create a milieu that could influence almost all phases of T-cell activation ranging from early activation to terminal functions, leading to CTL anergy and altering ICP availability (20). The great point is that CAFs selectively dysregulate or induce distinct iICPs on the tumor-infiltrated CD8+ T cells, and the pattern of induced ICPs is tissue specific. For example, TIGIT expression on the CD8+ T-cells decreased in the influence of pancreatic CAFs-derived secretome, while in reverse, melanoma CAF−derived factors containing l−arginase induce increased expression of TIGIT and BTLA in CD8+ T-cells (20).

In line with the interaction between inhibitory CAFs and TILs, IHC results of different tumor tissue showed T-cells in the TME (over 90% in some tissue) are localized to the stroma near SMA+ CAFs. In parallel with changes observed in CD8+ T cell (ICP over-expression), complementary changes occurred in CAFs (ICP ligands). Simultaneous up-regulation of iICPs (for example, BTLA) and corresponding ligands at the CAFs (for example, HVEM) leads to inhibition of CTLs in the tumor medium, which eventually leads to more inhibition of immune cells in the TME (20).

Sometimes inhibition of TILs by CAFs occurs through the interaction of CAFs with other immune cells. Likewise, the secretome of CAFs separated from hepatocellular carcinoma (HCC-CAF) leads to the recruitment and activation of neutrophils in TME *via* the SDF1a/CXCR4 signaling pathway (25). Neutrophils are one of the prominent components of solid tumors and are essential in cancer progression. Activation of neutrophils with HCC-CAFs conditioned media lead to less apoptosis, increased production of IL-8, CCL-2, and TNF-α by neutrophils, and over-expression of CD-66b and PD-L1 at their surface. PDL-1+ neutrophils can inhibit T-cell function through the IL-6-STAT3-PD-L1 signaling cascade, which provides a novel technique for activating and functioning neutrophils. Moreover, the role of HCC-CAFs in the induction of regulatory DC was approved, too. It was found that monocyte-derived DC adjacent with HCC-CAFs, compared with normal fibroblast co-culture, has higher expression of inhibitory molecules such as CD-14 and CTLA-4 but has lower expression of functional markers such as CD1a, CD83, and HLA-DR, CD80, and CD86, which this phenotype represents a tolerogenic DC. Subsequently, these educated tolerogenic DC induced compromised T-cell responses such as: decreased CD3+ T cell proliferation, increased differentiation of T-cell to CD4+ CD25+ Foxp3+ Treg, and lower IFN-γ expression by CD8+ T-cell (91).

Exosomes are cell membrane vesicles with a diameter less than 100 nm derived from the endosomal system. Accumulating evidence suggests that exosomes participate in TME cell communications through the horizontal transfer of information and, as a result, affect cancer cell proliferation and immunosuppression, and even the preparation of premetastatic niches in other organs. It has been identified that these micro-packages play an essential role in the interaction between CAFs and tumor cells (99). Dongwei Dou et al. using CAFs isolated from human breast cancer tissues, demonstrated that PD-L1 expression in breast cancer cells can be greatly boosted by exosomes produced from CAF and afterward induce decreased proliferation and increased apoptosis in T-cells and impaired NK cells function. As exosome-based miRNA delivery is a crucial CAFs’ strategy for influencing other TME cells, they compared miRNA profiles between CAF-derived exosomes and NF-derived exosomes obtained from breast tissue. They found that hsa-miR-92 is the most up-regulated miRNA in CAFs-derived exosomes that, following entry breast cancer cells, leads to enhancement of PD-L1 transcription, as well as to increase in migration, invasion, and proliferation of breast cancer cells (94). Another study that exposed NK cells to eradiated CAF cells isolated from NSCLC specified that CAFs decreased stimulatory receptors, cytotoxic capacity, degranulation rate, and proliferation of NK cells, and simultaneously increased the expression of inhibitory receptors on the surface of NK cells.

## Discussion

Anti-tumor immunity comprising the networks is generally designed to recognize and eliminate tumor cells. T lymphocytes are the key orchestrator of the host immune systems in TME, to the extent that the numbers and phenotypes of TILs are not only predictive of response to therapy but also correlate with clinical outcomes such as improved prognosis or DFS in various tumor types (74).

One of the essential regulatory mechanisms that inactivate penetrated TILs in cancer lesions is the induction and overexpression of iICPs or their ligands in TME. Following the breakthrough of immunotherapy based on the iICP blockers, a new era of cancer treatment has emerged that enables overcoming inhibitory signals of T cells and recovering cytotoxic function in TME (100). A collection of evidence showed promising clinical results and demonstrated effectiveness against a variety of malignancies like malignant melanoma and non-small cell lung cancer (101, 102).

But in the other hand, the shortcomings of iICP monotherapy suggest the need for a combination strategy. The potential of iICPs in combination with a TME-modulating agent for the treatment of tumors can be helpful, and the identification of the optimal combination treatment using iICPs as promising targets for next-generation immunotherapies is gaining extensive attention (20, 103).

CAFs, as the major constituents of the tumor stroma in many solid tumors, have pro-invasive and pro-metastatic activities and they are considered a tool and a potential target in cancer diagnosis and treatment, respectively (104). Stromal-activated fibroblasts participate in the regulation of both innate and adaptive anti-tumor immune responses and affect the trafficking, differentiation, and activation of different populations of immune cells, both locally and systemically (19).

From the point of view of the induction or expression of iICPs and suppression of immune response in TME, CAFs are key players, too. The data indicated that the expression of these inhibitory molecules is not due to *in vitro* conditions, but is mediated by phenotypic changes during fibroblast activation in TME. Actually, it is a part of education *via* the tumor to acquire tumor-promoting activities by CAFs (25).

The role of the CAFs soluble factors in the suppression of T cells activity is quite selective. As in melanoma was shown intracellular granzyme B levels in CD8+ T cells were decreased considerably in the presence of the melanoma-activated fibroblast secretome, while IFN-γ release was not affected. Concurrently, increased expression of TIGIT and BTLA in CD45RO+ memory cytotoxic T cells was seen, whereas the expression of PD-1, TIM-3, or LAG-3 on CD8+ T stayed unchanged (20).

Accordingly, broader comprehension of the functional mechanisms of CAFs and their interactions with the cancer cells, metastatic niche, immune cells, extracellular matrix, and endothelial cells could help design novel multi-targeted immunotherapies, especially for CAF-rich tumors such as PDAC. For example, previous studies showed some solid tumors might be made more susceptible to iICPs by arginase inhibition, and increase the efficiency of the PD-1/PD-L1 blockade (89).

Based on available studies, specific expression patterns of ICPs by CAFs during tumor development can be helpful in aiding diagnostic and therapeutic strategies. Although CAFs are present in almost all solid tumors, their phenotypes and subpopulations are different depending on the tissue and tumor stage (REF). According to CAFs heterogeneity and the special distribution of different subsets in tumors, a detailed characterization needs to confirm CAF subpopulations expressing each inhibitory marker (72). Moreover, the differential expression patterns of iICPs by CAFs and tumor cells should be considered and determined in each tumor type (105). For example, in ovarian tumors, stromal cells express B7-H3 at higher levels than tumor cells, while in head and neck tumors, CAFs don’t express this marker. These contradictory results emphasize further study on the role of CAFs in the ICPs induction in TME. Sometimes this distinctive expression pattern determines different outcomes in prognosis or treatment. For example, prostate cancer remission and immunological suppression are related to CD73 expression in the prostate epithelium. On the other hand, its expression in the stroma is related to a good prognosis for the disease (89).

Treatment of solid cancers based on CAFs has different strategies, including CAF-directed anti-cancer therapy, which means removing CAFs by complete deletion, and CAF-indirected anticancer therapy, which tries to perform stromal metabolic reprogramming or CAF normalization (means converting CAFs to NFs). The latter consist of techniques that change the state, phenotype, activity level, production, and signaling of activated fibroblasts. In this regard, one of the goals can be set as inhibition of iICP expression by CAF because NF, rather than CAF, has a lower level expression of iICPs (32, 106).

The abundance of CAFs, especially the FAP+ subset, has been clinically related to a poor prognosis in a number of cancers, including lung cancer, breast cancer, and CRC (74, 107). The expression of iICPs on the surface of these cells helps to explain why CAFs are related to poor patient prognosis and illustrate a new mechanism of T cell depletion and dysfunction within tumors (75, 108).

There is still little information on the interaction between immune cells and CAFs during and/or after radiation (19). Sometimes following treatments, such as radiotherapy or chemotherapy, we will have increased expression of inhibitory markers in the TME. On the other hand, according to the notion that radiation enhanced the pro-tumorigenic ability of CAFs as well as their ability to induction or expression of iICPs, it is necessary to determine crosstalk between CAFs and immune cells during and/or after radiotherapy.

## Author contributions

MR and FE designed the outline of the article, wrote the manuscript, and MR revised the paper, and approved the final manuscript. All authors contributed to the article and approved the submitted version.

## Funding

The present study was supported by grants from the Isfahan University of Medical Sciences, Isfahan, Iran [Grant No.140160].

## Conflict of interest

The authors declare that the research was conducted in the absence of any commercial or financial relationships that could be construed as a potential conflict of interest.

## Publisher’s note

All claims expressed in this article are solely those of the authors and do not necessarily represent those of their affiliated organizations, or those of the publisher, the editors and the reviewers. Any product that may be evaluated in this article, or claim that may be made by its manufacturer, is not guaranteed or endorsed by the publisher.
